# What is the value of reactive case detection in malaria control? A case-study in India and a systematic review

**DOI:** 10.1186/s12936-016-1120-1

**Published:** 2016-02-06

**Authors:** Anna Maria van Eijk, Lalitha Ramanathapuram, Patrick L. Sutton, Deena Kanagaraj, G. Sri Lakshmi Priya, Sangamithra Ravishankaran, Aswin Asokan, Nikunj Tandel, Ankita Patel, Nisha Desai, Ranvir Singh, Steven A. Sullivan, Jane M. Carlton, H. C. Srivastava, Alex Eapen

**Affiliations:** Center for Genomics and Systems Biology, Department of Biology, New York University, New York, NY 10003 USA; National Institute of Malaria Research Field Unit, Indian Council of Medical Research, National Institute of Epidemiology Campus, Ayapakkam, Chennai, Tamil Nadu India; National Institute of Malaria Research Field Unit, Civil Hospital, Nadiad, Gujarat India; Acsel Health, 500 5th Ave, Suite 2760, New York, NY 10110 USA

**Keywords:** Malaria, Reactive case detection, Surveillance, *Plasmodium falciparum*, *Plasmodium vivax*

## Abstract

**Background:**

Reactive case detection (RCD) for malaria is a strategy to identify additional malaria infections in areas of low malaria transmission and can complement passive surveillance. This study describes experiences with RCD in two Indian sites, and aimed to synthesize experiences with RCD across endemic countries.

**Methods:**

RCD programmes were piloted in two urban areas of India with a low prevalence of mainly *Plasmodium vivax* malaria in 2014. Cases were identified in a clinic by microscopy and contacts were screened within 2 weeks; PCR, in addition to microscopy, was used to detect *Plasmodium* parasites. A systematic review was conducted to identify RCD experiences in the literature.

**Results:**

In Chennai, 868 contacts were enrolled for 18 index cases of clinical malaria; in Nadiad, 131 contacts were enrolled for 20 index cases. No new malaria infections were detected in Nadiad among contacts, and four new infections were detected in Chennai (three *P. vivax* and one *Plasmodium falciparum*), of which two were among household members of index cases. An additional five studies describing results from an RCD strategy were identified in the literature: four in Africa and one in Thailand. Including the results from India, the average number of contacts screened per index case in a total of seven studies ranged from four to 50, and 126 in a case study in Thailand with one index case. Malaria was detected in 0–45 % of the contacted persons. The average number of index cases needed to be traced to find one new case of malaria ranged from one to five, and could not be assessed in one study in India (no contacts positive for 20 cases). Sharing the household with an index case was associated with a five-fold increased risk of malaria compared to contacts from households without an index case (pooled risk ratio 5.29, 95 % CI 3.31–8.47, I^2^ 0 %, four studies).

**Conclusions:**

RCD in areas of low malaria transmission is a labour-intensive strategy, and its benefit is not clear. Studies are needed to assess how RCD can be optimized or into alternatives where interventions are targeted to family members or hotspots.

## Background

Malaria elimination returned to the global agenda in 2007, stimulating a surge in malaria control and elimination efforts in the remaining malarious countries in the world [1]. The resulting boost in the use of insecticide-treated nets and effective malaria treatment has led to remarkable reductions in the incidence of malaria in many countries or regions [2]. Given the drastic reduction of malaria, some malaria-endemic countries are now faced with the question of how to fully eliminate the remaining cases. For countries with a low malaria prevalence (<5 %) robust and responsive surveillance systems are critical for the success of malaria control and elimination [3]. Ideally, in elimination settings, surveillance must be an intervention in which immediate action is taken in response to case identification [3]. Reactive case detection (RCD), the process of identifying further cases following the identification of a locally transmitted case, is one of the strategies which has been advocated in these circumstances [4]. The use of RCD is based on the observations of malaria hotspots, a clustering of cases in space and time which can feed malaria transmission throughout the year [5, 6], and the assumption that asymptomatic malaria can be present across the malaria spectrum, and is higher in households of identified clinical cases and in their neighbourhood [7, 8]. The presence of asymptomatic malaria may be dependent on the speed at which malaria transmission decreases: when the decrease in transmission is more rapid than loss of immunity in a population, the reservoir of asymptomatic carriers can be significant [4]. Some have stressed the importance of RCD in the dry season to reduce the reservoir of infections before the rainy season [7].

**Fig. 1:**
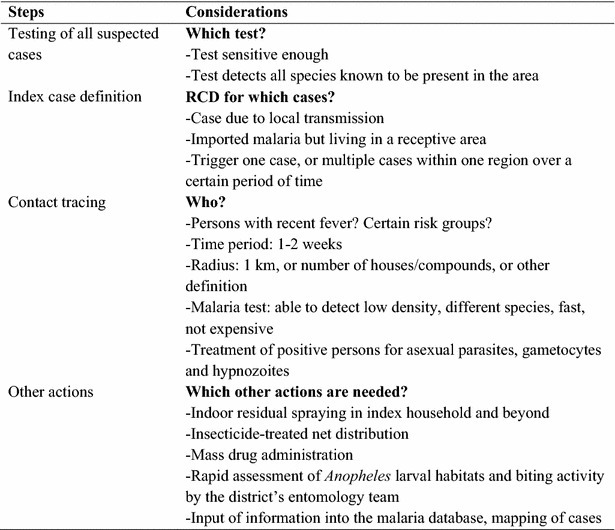
Steps in RCD. Sources: WHO 2012 Disease surveillance for malaria elimination: an operational manual [4]; Zanzibar malaria control programme 2009 Malaria elimination in Zanzibar: a feasibility assessment [26]; Smith Gueye et al. [10]

There is widespread confusion in the terms of [9], and a wide range in approaches to, RCD [10]; a summary of potential steps is presented in Fig. 1. A survey among 13 countries in the Asia–Pacific region with national or sub-national malaria elimination goals showed there is considerable variety in the practice of case investigation [10], the trigger typically being a single case report or a defined threshold of multiple cases. The spatial range of screening can vary from a specific number of households to an entire administrative unit (e.g., village) but the optimal radius is unclear [10]. The strategy is labour-intensive, and expensive; in addition, the common detection methods, microscopy or a rapid diagnostic malaria test, can miss low-density infections that are still capable of transmitting malaria [11]. There is very little information on how RCD programmes work in practice, if they achieve their goal, and if they are cost-effective, with little evidence to guide practice.

Malaria transmission in India is diverse, with both *Plasmodium falciparum* and *Plasmodium vivax* present, and transmission levels vary from high in the northeast with a predominance of *P. falciparum* to low in most of the country with a predominance of *P. vivax*. As part of an ongoing investigation into the epidemiology of malaria in India [12], clinic surveys and RCDs were conducted simultaneously at two sites in 2014 for the Center for the Study of Complex Malaria in India [13]. The aims of the current analyses were to describe experiences with RCD in these two urban sites with mainly *P. vivax* transmission, and to synthesize experiences with RCD from the literature in order to assist with the development of an evidence-based framework for the future use of RCD.

## Methods

### Case studies

#### Setting

The case studies were conducted in Chennai and Nadiad. Chennai, the capital of the southern state of Tamil Nadu, is located on the coast of the Bay of Bengal and had a population of ~4.7 million and a population density of 26,903/sq km in 2011 [14]. The climate in Chennai is categorized as ‘tropical wet and dry’, with temperatures ranging from ~15 °C (January) to ~45 °C (May) and a relative humidity between 59 and 80 %. Monsoons come in two waves: the main rainfall period is from October–December as part of the northeast monsoon, but some rains also come during the southwest monsoon between July–August [15]. Malaria transmission (predominantly *P. vivax*) in Chennai city is perennial and peaks between July and October. The Besant Nagar Malaria Clinic is attached to the Regional Office of Health and Family Welfare of the Government of India in a predominantly residential neighbourhood in Chennai composed of middle- and upper-class dwellings, with a few slums and a large coastal fishing community. Nadiad town is located in the Kheda district in the central part of Gujarat State and has a population of ~225,000. Nadiad has a sub-tropical and semi-arid climate, receiving the majority of its annual precipitation during the southwest monsoon season (June–September) [15]. Malaria endemicity is considered hypo-endemic, with *P. vivax* and *P. falciparum* prevalence rates oscillating throughout the year based on the transmission season. The National Institute of Malaria Research (NIMR) Malaria Clinic is located in the Civil Hospital of Nadiad in a predominantly residential neighbourhood.

#### Procedures

Index cases (positive for malaria by microscopy) were identified in the respective malaria clinics. Only persons aged 1–70 years and without severe anaemia were eligible for enrolment; pregnant women were excluded. After informed consent, a structured questionnaire was completed on sociodemographics, history of malaria, use of malaria prevention, and clinical information. The household of the index case was visited within 1–2 days of the identification of the index case, and coordinates were recorded using a global positioning system. The same questionnaire as for the index case was used for subjects in the index household after consent, and blood was obtained by finger-prick for microscopy, Hemocue^®^ and polymerase chain reaction (PCR). A door-to-door fever survey was done in proximal households (households residing in the index apartment complex or within 100 m of index case house) and distal households (households within 100–1000 m of the index case in Nadiad and within 200 m of the index case in Chennai due to the high population density), whereby persons with fever (documented fever or a history of fever in the last 2 weeks) and a proportional number of asymptomatic persons (every third to fourth household) were enrolled within 1–14 days of the index case, using the same procedures as described above. Participants with a positive malaria test were treated as per national guidelines (*P. vivax*: chloroquine 25 mg/kg over 3 days and primaquine 0.25 mg/kg for 14 days; *P. falciparum* artesunate 4 mg/kg for 3 days in combination with sulfadoxine 25 mg/kg and pyrimethamine 1.25 mg/kg on the 1st day and primaquine 0.75 mg/kg). All participants were given contact information in the event that symptoms would arise within a 2-week period.

#### Laboratory tests

Haemoglobin level was assessed using Hemocue (HemoCue, Ängelholm, Sweden). Thin and thick smears were stained using Giemsa and at least 300 fields in the thick smear were examined using the 100× oil immersion before a slide was called negative for malaria. Parasites were counted on the thick smear against 200–500 leukocytes. The results were expressed as parasites per µl of blood, using the white blood cell (WBC) count if known, or assuming 8000 WBC/µl blood. At each site, slides were routinely read by two microscopists, and a third microscopist was used if the results were disparate. All samples, whether positive or negative by microscopy, underwent DNA extraction by QIAamp DNA blood Mini Kits (Qiagen Inc, Valencia, CA, USA). A modified nested, multiplex-PCR method targeting the 18S small sub-unit ribosomal protein (SSU rDNA) was used for species-specific detection of *Plasmodium* parasites [16, 17]. Amplicons were visualized on a 1.5 % agarose gel, and fragment sizes differentiated using a 100 bp DNA ladder (exACT gene, Thermo Fisher Scientific, Pittsburg, PA, USA).

#### Analysis

Documented fever was defined as an oral temperature of 37.5 °C or more. A secure, web-based application REDCap (Research Electronic Data Capture) database was used to capture and store all subject data and test results [18]. Subsequently, data were exported into Stata (Stata/IC version 13.1, StataCorp LP, College Station, USA) for analysis. Characteristics of index cases and participants of the RCD study were compared using the Fisher exact test for 2 by 2 tables and the *t* test for continuous variables.

#### Ethical approval

These studies received ethical approval from the Institutional Review Board at New York University School of Medicine and the Human Subjects Ethical Committee at the National Institute of Malaria Research (ICMR) in New Delhi.

#### Systematic review

PubMed database, Google Scholar and reference lists were searched to identify studies with information on RCD up to October 2015 using the search terms “malaria and (contact tracing OR focal screening OR reactive case detection OR reactive case investigation)” [9]. Studies were eligible if they reported results of an RCD strategy. Information was extracted on location, time period, study design, and details of the RCD, such as number of cases followed, number of persons traced, and number of new cases detected. The prevalence of malaria among the screened persons, average number of clinical cases which needed to be followed to identify one new malaria case, and the average number of persons to be screened to identify one new malaria case were calculated, and the information was tabulated together with the results from the Indian RCDs. The last search was conducted on 21 September 2015. Where possible results were pooled using meta-analysis (metan procedure in Stata), including the data from India.

## Results

### Case study: Chennai

**Fig. 2:**
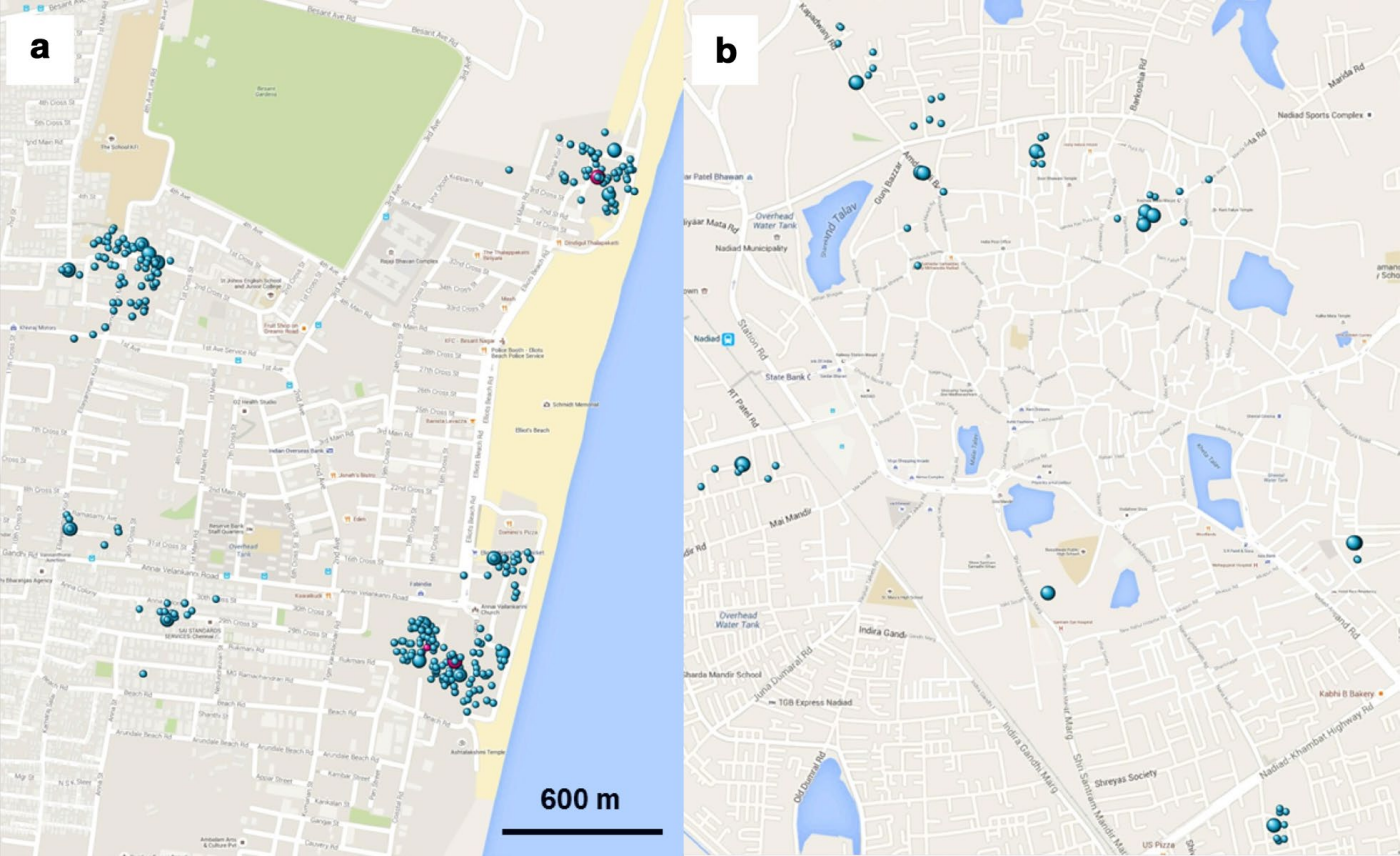
Mapping of RCD clusters. Representative blocks (~3.6 sq km) of the RCD areas of **a** Chennai and **b** Nadiad are shown. *Large spheres* index case households; *small spheres* proximal and distal RCD households; *red* malaria-positive reactive cases, *blue* negative

**Fig. 3:**
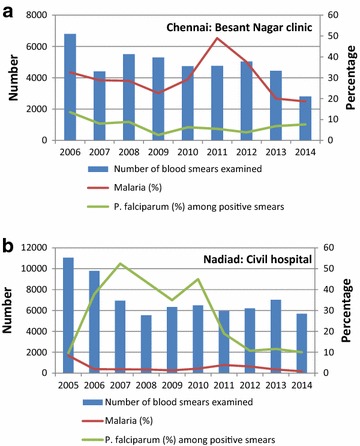
Malaria cases over time in the clinics where the index cases were recruited. **a** Chennai, Tamil Nadu, India, **b** Nadiad, Gujarat, India

In Chennai, 18 out of 60 malaria cases (30.0 %) in the study period consented as an index case for participation in the RCD. The number of participants recruited per index case ranged from 13 to 74 (median 53), with a total of 868 persons examined, of whom 126 (14.5 %) had a history of fever or documented fever (Table 1; example of distribution in Fig. 2a). Eighty-three participants (9.6 %) shared the household with the index case with recruitment of 91.6 % of them within 1st week; 191 (22.0 %) and 594 (68.4 %) were proximal and distal participants, respectively, with recruitment within 2 weeks of 93.2 and 55.7 %. There were significantly more women among the RCD participants (p = 0.001) and RCD participants were less likely to have salaried employment compared to the index cases (p = 0.02). In addition, RCD participants were less likely to have had malaria in the past year compared to the index case (p = 0.006). Four participants traced from four different cases were positive by both PCR and microscopy: three for *P. vivax* (one in same household and two in proximal households) and one for *P. falciparum* (in same household as the index case; p = 0.096 for same *versus* proximal or distal household). No mixed infections or infections with other species were detected. All malaria-positive participants had a history of fever but documented fever was not detected and none had taken anti-malarials in the past 2 weeks. Two *P. vivax* cases were detected in the dry season, and one *P. vivax* case and the *P. falciparum* case were detected in the rainy season. Gametocytes for *P. falciparum* were not detected, whereas gametocytes for *P. vivax* were detected in all three RCD participants positive for *P. vivax.* Routine data from the same clinic as the index cases showed that there was an increase in malaria in 2010–2012, but the prevalence has decreased since then with *P. vivax* as the predominant species, and *P. falciparum* contributing to <10 % of malaria infections (Fig. 3a).

**Table 1:** Characteristics of participants by location and type of study, India, 2014

	Chennai		Nadiad	
	Index cases N = 18	RCD N = 868	Index cases N = 20	RCD N = 131
Time period	Jan 14–Dec 14	Jan 14–Jan 15	Feb 14–Aug 14	Mar 14–Sep 14
Mean age, 95 % CI, years	32.3, 25.5–39.0	31.5, 30.5–32.6	27.3, 19.4–35.2	37.9, 35.0–40.8^a^
Age <18 years (%)	3 (16.7)	171 (19.7)	7 (35.0)	16 (12.2)^a^
Female (%)	5 (27.8)	526 (60.6)^a^	6 (30.0)	78 (59.5)^a^
Recruited in rainy season (%)	10 (55.6)	472 (54.4)	9 (45.0)	60 (45.8)
Among persons ≥18 years	N = 15	N = 697	N = 13	N = 115
Primary school highest level (%)	8 (53.3)	230 (33.0)	6 (46.2)	46 (40.0)
Salaried employment (%)	9 (60.0)	187 (26.1)^a^	4 (30.8)	12 (10.4)
History and symptoms	N = 18	N = 868	N = 20	N = 131
Documented fever (%)	7 (38.9)	20 (2.3)^a^	11 (55.0)	7 (5.3)^a^
History of fever in last 48 h (%)	18 (100)	121 (13.9)^a^	9 (45.0)	27 (20.6)^a^
History of or documented fever (%)	18 (100)	126 (14.5)^a^	16 (80.0)	29 (22.1)^a^
History of malaria in last year (%)	5 (27.8)	48 (5.5)^a^	2 (10.0)	3 (2.3)
Anti-malarials used in last year (%)	5/5 (100.0)	31/47 (63.8)	0/2 (0.0)	0/3 (0.0)
Use of nets^b^ (%)	1 (5.6)	21 (2.4)	1 (5.0)	16 (12.2)
Use of mosquito repellent (mat/vapour/coil)	4 (22.2)	279 (32.1)	5 (25.0)	56 (42.8)
History of travel in last 14 days (%)	2 (11.1)	114 (13.1)	1 (5.0)	8 (6.1)
Anaemia (%)^c^	5 (27.8)	316/866 (36.5)	14 (70.0)	87 (66.4)
Mean haemoglobin, 95 % CI, g/dl	12.9, 11.7–14.1	12.6, 12.4–12.7	11.0, 9.8–12.1	11.4, 11.1–11.7
Laboratory result	N = 18	N = 868	N = 20	N = 131
Microscopy: any species (%)	18 (100)	4 (0.5)	20 (100)	0
*P. falciparum*	3 (16.7)	1 (0.1)	1 (5.0)	0
*P. vivax*	15 (83.3)	3 (0.4)	19 (95.0)	0
Gametocytes *P. falciparum* (%)	1 (5.6)	0	1 (5.0)	0
Gametocytes *P. vivax* (%)	15 (83.3)	3 (0.4)	19 (95.0)	0
Parasites densities 95 % CI, per μl				
*P. falciparum*	843, 34–19,253 (n = 3)	800 (n = 1)	3160 (n = 1)	–
*P. vivax*	1850, 934–3664 (n = 15)	1754, 517–5950 (n = 3)	3816, 2280–6388 (n = 19)	–
Gametocytes *P. falciparum*	40 (n = 1)	–	520 (n = 1)	–
Gametocytes *P. vivax*	860, 498–1484 (n = 15)	395, 160–972 (n = 3)	1496, 832–2692 (n = 19)	–
PCR: any species (%)	17/17 (100.0)	4 (0.5)	17 (85.0)	0/131 (0.0)
*P. falciparum*	3 (16.7)	1 (0.1)	1 (5.0)	0
*P. vivax*	14 (77.8)	3 (0.4)	16 (80.0)	0

*PCR* polymerase chain reaction, *RCD* reactive case detection

^a^p < 0.05 comparing index cases versus reactive case detection participants in the same location

^b^Nets mainly untreated (99 %)

^c^Age and gender appropriate definition of anaemia (<11 g/dl if < 5 years, <11.5 g/dl if 5–11 years, <12 g/dl if 12–14 years or >15 years and female, <13 g/dl if male and >15 years) [40]

### Case study: Nadiad

In Nadiad, 20 out of 42 malaria cases (47.6 %) in the clinic in the study period consented as index case for participation in the reactive case study. The number of participants recruited per index case ranged from 2–13 (average and median 6.5), with a total of 131 persons, of whom 29 (22.1 %) had a history of fever or documented fever. Thirty-two participants (24.4 %) shared the household with the index case with recruitment of 84.4 % of them within one week: 60 (45.8 %) and 39 (29.8 %) were proximal and distal participants, respectively, with recruitment within 2 weeks of 93.3 and 94.9 % (example of distribution in Fig. 2b). Index cases were significantly younger (p = 0.03) and more likely to be male (p = 0.03) compared to the RCD participants. None of the RCD participants was positive by any malaria test, and no gametocytes were detected by microscopy. Routine data from the same clinic as the index cases showed that malaria prevalence was low (<4 %), and the proportion of *P. falciparum* infections was decreasing (Fig. 3b).

### Systematic review

**Fig. 4:**
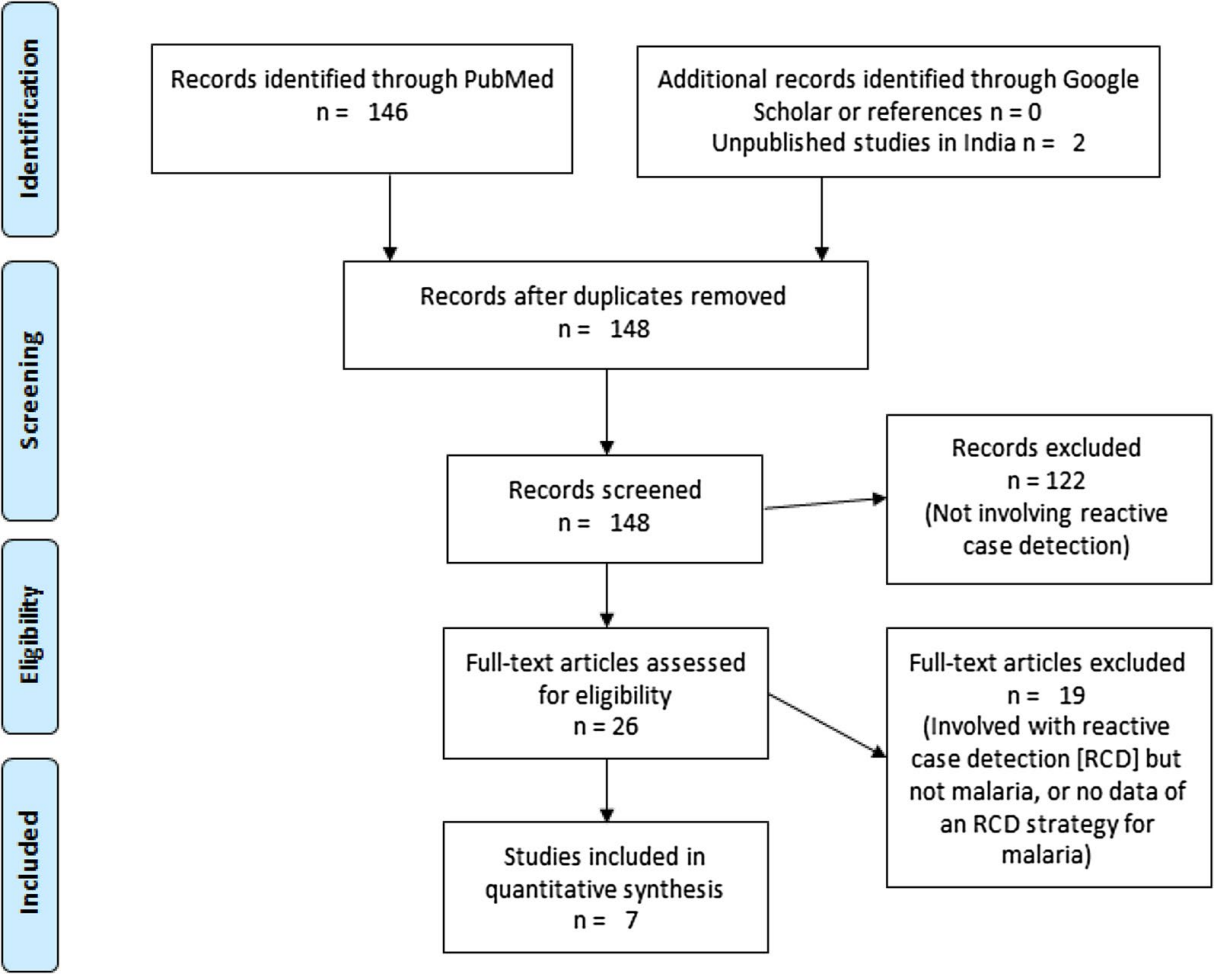
Flow diagram for systematic review

**Fig. 5:**
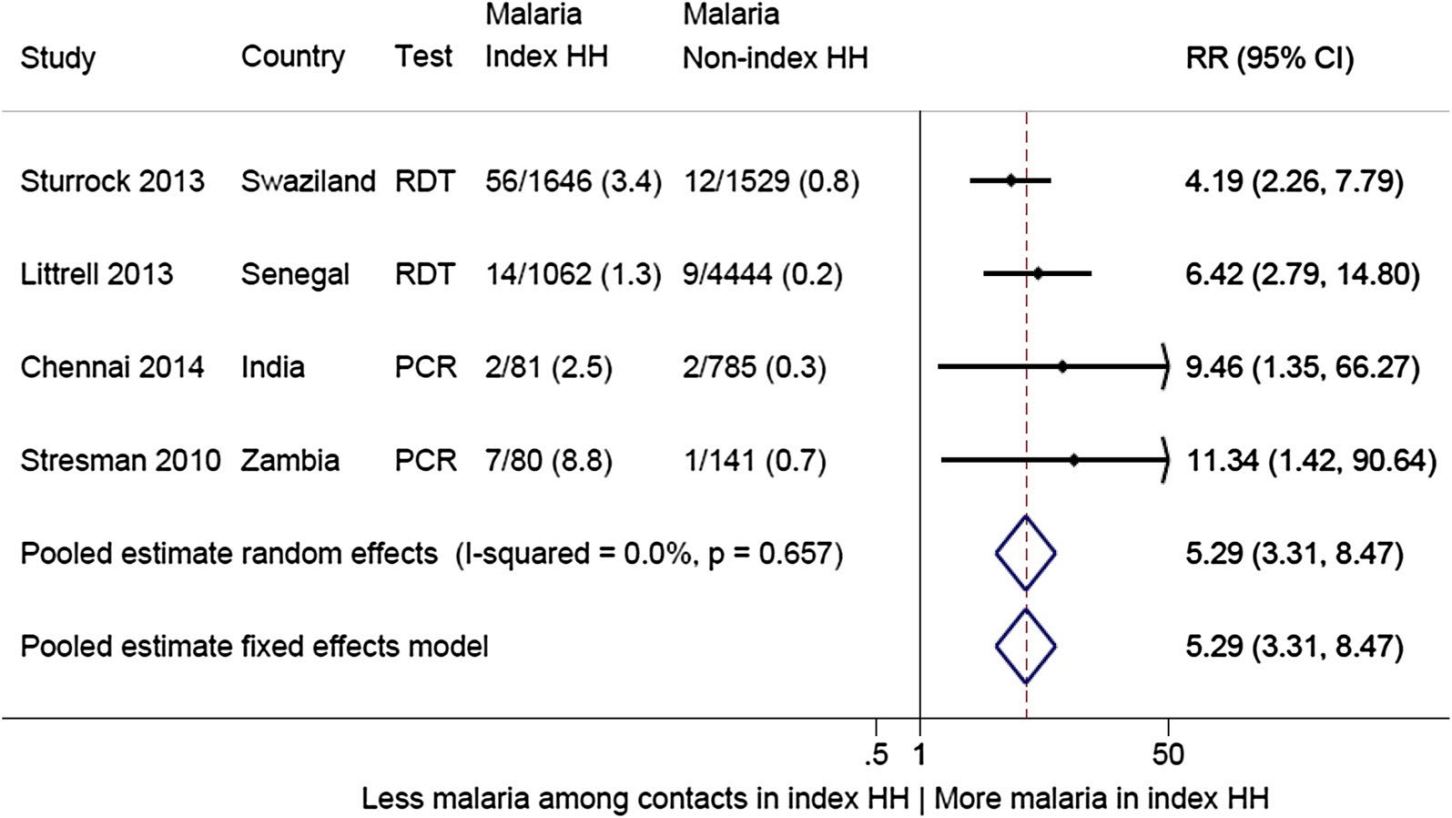
Comparison of malaria detected during contact tracing among members of index case households *versus* among members of other (more distal) households, studies in Africa and India, 2009–2015. This is an analysis where the raw numbers have been used, and no adjustment was done for clustering at the household level or by index case or other factors. In the study by Stresman et al., the non-index households were randomly selected from the same locality [19]. CI confidence interval, HH household, PCR polymerase chain reaction, RDT rapid diagnostic malaria test, RR risk ratio

Of the 146 articles retrieved from PubMed, five studies contained results of an RCD strategy (Table 2; Fig. 4): four were conducted in sub-Saharan Africa in areas with *P. falciparum* and one in Thailand with both species present [19–23]. Searches in Google Scholar and through references of identified studies did not yield new information. Including the RCDs from India, the number of index cases examined varied from one in Thailand to 426 in Zambia, and number of contact persons screened ranged from 131 to 5520, with the average number of contacts per index case ranging from four to 126 (Table 2). Three studies used RDT only to test the contacts, one study reported PCR and RDT, and three studies used microscopy and PCR; the last three were all in Asia (Table 2). Only one study in Senegal reported a participation rate (98 %), whereas all other studies did not provide numbers but one study in Swaziland noted “a tendency to just screen the index household”. Four studies had information regarding the actual timeline of recruitment of contacts, and the majority of participants were recruited within 2 weeks [22, 23] (Table 2). The prevalence of malaria among contacts was very low (0–3.4 %), except for one study in Zambia where a prevalence of 45.3 % was detected [20]. For four studies, information could be pooled for the comparison of malaria among household members of the index case *versus* members of other households (Fig. 5). Household members of index cases were overall five times more likely to have malaria detected than members of other households (pooled risk ratio 5.29, 95 % CI 3.31–8.47, using unadjusted estimates). The I^2^ was 0 %, indicating that all variability in the risk ratio estimate was due to sampling error within studies and not to heterogeneity between studies [24]. However, this proportion must be considered with caution given the small number of studies.

**Table 2:** Review of results of RCD studies as detected in medical literature and results from India, September 2015

Study	Stresman 2010	Pinchoff 2015	Littrell 2013	Sturrock 2013	Rogawski 2013	Chennai 2014	Nadiad 2014
Country, region	Zambia, Choma and Namwala district	Zambia, Chongwe district	Senegal, Richard Toll District	Swaziland (national data)	Thailand, Bo Rai district	India, Chennai, Tamil Nadu	India, Nadiad, Gujarat
Time period	Jun–Aug 2009 Dry season	Jun 12–Jun 13 All seasons	High transmission season 2012	Dec 09–Jun 12 All seasons	July 2011	Jan 14–Jan 15. All seasons	Mar 14–Sep 14. All seasons
Malaria transmission	Seasonal transmission, Pf_6–59m_ 7.9 % Central province (MIS 2008)	Seasonal transmission, Pf_6–59m_ 0.0 % Lusaka Province (MIS 2012)	Seasonal transmission, Pf_6–59m_ 0.7 % North Region (DHS 2012–2013)	Seasonal transmission, all age groups: 0.07 % (MIS 2010)	Low and seasonal	Low and seasonal	Low and seasonal
Main species	*P. falciparum*	*P. falciparum*	*P. falciparum*	*P. falciparum*	*P. vivax* + *P. falciparum*	*P. vivax* + *P. falciparum*	*P. vivax* + *P. falciparum*
Cases	4 Rural clinics	1 Clinic	13 Clinics	Health facilities in Swaziland	1 Hospital	1 Urban clinic	1 Urban clinic
Trigger and malaria test	Case (RDT) in clinic	Case (RDT and microscopy) in clinic	Case in clinic (test not reported)	Locally acquired cases or imported cases if local ecology is able to support transmission (test not reported)	1 case in hospital (tests not reported)	Case in clinic (microscopy)	
Spatial extent	Homestead of index case. Fever was not a criterion	All household members. Fever was not a criterion	Index case compound and 5 neighbouring compounds. Fever was not a criterion	Index case household and neighbouring households within 1-km radius	Neighbours within 1 km of index case, fever was not a criterion	All household members of the index case, persons with a history of fever and a sample of persons without fever in proximal (same apartment block) and distal households (within 0.2 km)	
Time line response per protocol	Within 2 weeks	Not reported	Within 3 days of case	Not reported	Not reported	Household within 1–7 days, proximal and distal: within 14 days	
Time line response in reality	No information	No information	No information	48.6 % screened within 1 week, 87.3 % screened within 14 days. Range 0–90 days	After 2 weeks	Household members: 91.6 % screened within one week. Others: 64.8 % within 2 weeks. Range: 0–32 days	Household members: 84.4 % screened within one week. Others: 93.9 % within 2 weeks. Range: 2–28 days
No of Index cases	23	426	110	247	1 (Pf and Pv)	18 (3 Pf, 15 Pv)	20 (1 Pf, 19 Pv)
<5 years (%)	Not reported	185 (43.4 %)	1 (0.9)	Not reported (mean 25.9 years)	Not reported	0 (0 %)	1 (5.0 %)
Male (%)	Not reported	228 (53.5 %)	90 (81.8 %)	151 (61.1)	Not reported	13 (72.2 %)	14 (70.0 %)
No of contacts screened	186	1621	5520	3671	126	868	131
<5 years (%)	46 (24.7 %)^a^	Not reported	1021 (18.5 %)	163 (4.4 %)	Not reported	44 (5.1 %)	2 (1.5 %)
Male (%)	86 (46.2 %)	Not reported	2782 (50.4 %)	2168 (59.1 %)	Not reported	342 (39.4 %)	53 (40.5 %)
Average contacts/case	8 (range 3–19)	4 (range 0–13)	50 (range not reported)	15 (range 1–157)	126	48 (range 13–74)	7 (range 2–13)
Positive among contacts	RDT: 5/185 (2.7 %). PCR: 7/186 (3.4 %)	RDT: 735/1621 (45.3 %)	RDT: 23/5520 (0.4 %)	RDT: 74/3671 (2.0 %)	Microscopy: 1/126 (0.8 %, Pf). PCR: 2/126 (1.6 %, 1 Pf, 1 Pv)	Microscopy and PCR: 4/868 (0.5 %, 3 Pv, 1Pf)	Microscopy and PCR: 0/131 (0.0 %)
Average number of index cases needed to be traced to find one new case^b^	5	1	5	3	Not applicable	5	>20
Average number of contacts needed to be screened to find a new case^c^	37	2	240	50	126 (microscopy in duplicate) 63 (PCR)	216	>140

*PCR* polymerase chain reaction, *Pf* *Plasmodium falciparum*, *Pv* *Plasmodium vivax, RCD* reactive case detection, *RDT* rapid diagnostic test

^a^≤5 years of age

^b^Number of index cases divided by number of new cases detected

^c^Product of number of index cases to be screened for one new case times the average of contacts per index case

Several other important associations were reported in individual studies. For instance, one study in Zambia reported on gametocyte rate of *P. falciparum* using PCR and detected gametocytes in 2.4 % (2/87) of index case household members and 0 % (0/141) among other contacts (p = 0.145) [19]. The study in Senegal detected a higher prevalence of malaria among contacts with a travel history (13/81 or 16.0 % *versus* 10/5437 or 0.2 %, p < 0.001) [21]. The study in Swaziland reported significant higher odds of malaria among contacts within the 1st week from presentation of the index case compared to more than 2 weeks, and reduced odds if the index house was sprayed [22]. In the study in Thailand, PCR identified four cases which were missed by routine microscopy [23]. Finally, the study in Zambia with a high prevalence of malaria reported that household contacts were significantly more likely to be positive when the index case was <5 years old, and with increasing distance from the main road [20].

## Discussion

RCD is a strategy recommended to reduce malaria in areas of low prevalence [4]. In two urban areas of India with mainly *P. vivax* malaria, RCD in 2014 resulted in the detection of 0.5 % malaria cases in Chennai, and none in Nadiad. An additional five studies describing results from an RCD strategy were identified in the literature: four in Africa and one in Thailand. Including the results from India, the average number of contacts screened per household ranged from four to 50, and was 126 in a case study in Thailand with one index case. Malaria was detected in 0–45 % of the contacted persons. Sharing the household with an index case was associated with a five-fold increased risk of malaria using unadjusted information from four studies.

### Case studies in India

Using RCD, no new cases were identified in the field site in Nadiad, Gujarat, and few new cases in Chennai, Tamil Nadu. Travel and use of repellents was no different among index cases compared to contacts in both sites; the use of anti-malarials was low, and there was a negligible use of insecticide-treated nets. A sensitive test (PCR) was used to detect the species of malaria parasite, which made it less likely that infections of low density would have been missed. The sample size in Nadiad was low given the population density, and study staff reported problems in screening contacts, such as the absence of household members at the time of visit and obtaining consent from potential participants. Unfortunately the number of refusals was not recorded in both sites, and neither was a census available to estimate the number of people in the area that should have been approached. An alternative explanation is that the prevalence of malaria has been low in Nadiad for a considerable time, and infections become clinical and are immediately detected, or are a result of exposure during travel. Figure 3b confirms the low prevalence of malaria in this area over the past 10 years. In Chennai, entomological investigation of the increase in malaria in 2011 showed that the vector mosquito preferred overhead water tanks to open wells as breeding site [25]. Although both Chennai and Nadiad might qualify for an RCD strategy from a malaria control perspective given the low prevalence of malaria, in practice this proved very difficult [4]. The high population density made optimal coverage extremely challenging in the time that was allotted; in Chennai three staff and in Nadiad four staff were occupied full time with RCD. Staff encountered problems when screening contacts, such as the absence of household members at the time of visit (children to school, people at work elsewhere), people in a hurry with little time to participate in a research study, traffic congestion, a high density of people in small areas, and outside temperatures exceeding 37 °C. The strategy used (screening of only a proportion of non-fever cases and the absence of household members during the work day) may have resulted in missing additional malaria cases, however, a cost-benefit analysis may not favour a more intense approach, given that it is very likely that *P. vivax* hypnozoite-infected cases will be missed.

### Systematic review of RCD

In the studies considered in the review, it was not always clear what method of diagnosis was used to declare an index case. The assessment whether an index case was local or imported was only clearly described in the programme in Swaziland, where a choice was made to conduct RCD for imported cases and for cases in areas receptive to ongoing transmission [22]. Most studies chose to screen contacts independent of the presence of a complaint of fever of the contacts. Two studies only examined homesteads or household members of index cases, whereas two others used the criterion of within 1-km radius of the index household (Table 2). A 1-km radius definition is used by the World Health Organization based on the flight range of *Anopheles* mosquitoes, which is typically limited to 1–2 km [4]. In the study in Chennai, a shorter radius of 0.2 km was chosen because of the extremely population-dense urban setting, where index cases were frequently found in apartment blocks or adjacent houses consisting of multiple family homes. In the elimination feasibility analysis of Zanzibar, screening of approximately 100 households per case was suggested [26]. The study in Swaziland reported that “A 1-km screening radius appeared to be logistically challenging and may not be feasible in such resource-limited settings such as Swaziland” [22]. One study suggested the use of tablets loaded with satellite images from the area involved to estimate the number of households that should be screened [22]; although this may be valuable approach in a rural area, in an urban or semi-urban area this may be of limited value. Timing of the screening is important, as shown in the study in Swaziland where more contacts were positive in the 1st week after detection of the symptomatic index case compared to after 2 weeks [22]; however, except for household members, screening within 1 week was not optimal in three studies which presented information (Table 2). Several studies explored whether tightening the screening definition would have helped to identify contacts with asymptomatic malaria [27], and in Senegal the application of a restriction to screen persons with recent fever and/or travel might have reduced the amount of work [21]. One study in Zambia was conducted in a region where malaria is low according to the malaria indicator survey but the study recovered many infections when screening households of clinical cases (no other households were screened in this study), and a population-based approach may be more appropriate in such settings [20].

Limitations to the review included the inconsistent reporting and different methodologies of the case studies in the literature. In addition, the search may not have identified all relevant case studies of RCD. Finally, unadjusted risk estimates were used in the meta-analysis as adjusted risk estimates were not available in two studies because numbers were too small. In one study, taking clustering at the household level and by index case into account increased the malaria risk of a household member of an index case compared to a member of other households, whereas in another study the adjustment for travel history decreased this risk.

### RCD in the context of the literature

Organizations stress the importance of a state-of-the-art surveillance system for malaria [3, 28], but the best and most cost-effective strategy to deal with the results seems less clear, and that may also depend on the political will and amount of funding that a country can spend on malaria elimination. Many countries report an RCD component as part of their national programme [10, 29–32], but it is a highly labour-intensive strategy; except for the study by Pinchoff et al. in Zambia [20], three or more index cases and the screening of ~40 or more contacts per case was the minimum to detect one additional case of malaria in the studies examined. Currently there is no evidence that an RCD strategy impacts on malaria transmission in a malarious area; however, it is not clear how to evaluate the outcome of an RCD, and what would happen in its absence [33]. A modelling study in Zambia suggested that “the efficiency of this strategy is likely to decrease with declining parasite prevalence” [27]. Although programmes report that RCD is incorporated into their strategy, in practice this may not always be the case, as was reported from Indonesia: “Further, discussion showed that the Municipal Health Authority infrequently carried out epidemiological investigations of malaria cases in collaboration with staff from primary health care facilities” [31]. In Sri Lanka, RCD became more important as malaria declined and the programme instituted case investigation reviews in 2009 where each case and the follow-up measures taken were reviewed in detail by the central and regional malaria officers [29]. They reported: “Although coverage is relatively low, RCD is believed to help reduce the magnitude of peaks during transmission seasons by identifying both asymptomatic and symptomatic infections” [29]. In Mauritius, 27 % of an average of 36 cases per year between 2005 and 2008 were detected by RCD [32]. The value of RCD in an area with *P. vivax* transmission is not clear; gametocytes of *P. vivax* generally appear at the same time as asexual parasites but can be infectious to the mosquito before detection [34]. The hypnozoites of *P. vivax* can become activated weeks, months or years after infection and are not cleared unless a long course of primaquine is given. The risk of relapsing malaria following a *P. vivax* infection varies worldwide; both frequent-relapsing and long-latency strains are present in India [35]. There are additional limitations to an RCD strategy as summarized by Sturrock et al.: hotspots of purely asymptomatic malaria will be missed when using clinical malaria as starting point, in addition to hotspots among populations with low access to healthcare, or a false negative test among the index case, or if areas are considered unreceptive to malaria transmission by the programme [22].

Further studies into the efficacy of RCD are clearly needed. Studies into the cost-effectiveness of RCD are ongoing in Indonesia and Thailand [36]. Alternatives to RCD are also being explored, for example, the use of serology to detect *Plasmodium* antibodies for the identification of malaria hotspots [37]. Targeting hotspots with malaria interventions could potentially be more effective, and a trial in Kenya is currently examining this strategy [5]. A higher risk of malaria among household members of index cases was identified in the meta-analysis. Targeting household members of the index case with either treatment and/or insecticide-treated nets could be an alternative and less labour-intensive strategy and presumptive malaria treatment of household members of a symptomatic malaria case is currently being tested in The Gambia [38]. Some countries used repeated surveys in a limited, well-defined, at-risk population to detect and treat remaining malaria, and this may be appropriate in certain settings [39].

## Conclusions

RCD was not a useful strategy in two sites in India. It is important that countries document their experiences with RCD, so other countries can learn from them and the information can be used in modelling studies, which may lead to improved guidelines. In this way, RCD can find its niche in the current arsenal of tools to control, reduce or eliminate malaria.
